# Catheter Ablation of Tachyarrhythmias in Small Children

**Published:** 2005-01-01

**Authors:** Andrew D Blaufox

**Affiliations:** Children’s Heart Program of South Carolina – MUSC

## Introduction

An estimated 80,000-100,000 radiofrequency ablation (RFA) procedures are performed in the United States each year [1]. Approximately 1% of these are performed on pediatric patients at centers that contribute data to the Pediatric Radiofrequency Registry [2].  Previous reports from this registry have demonstrated that RFA can safely and effectively be performed in pediatric patients [3-4].  However, patients weighing less than 15 kg have been identified as being at greater risk for complications [3-4]. Consequently, there has been great reluctance to perform RFA in small children such that children weighing less than 15 kg only represent approximately 6% of the pediatric RFA experience [2]. despite the fact that this age group carries the highest incidence of tachycardia, particularly supraventricular tachycardia (SVT) [5].  Factors other than the risk of complications contribute to the lower incidence of RFA in this group, including the natural history of the most common tachycardias (SVT), technical issues with RFA in small hearts, and the potential unknown long-term effects of RF applications in the maturing myocardium.  Conversely, there are several reasons why ablation may be desirable in small children, including greater difficulties with medical management [6-8], the higher risk for hemodynamic compromise during tachycardia in infants with congenital heart disease (CHD), and the inability of these small children to effectively communicate their symptoms thereby making it more likely that their symptoms may go unnoticed until the children become more seriously ill.  Before ultimately deciding that catheter ablation is indicated in small children, one must consider which tachycardias are likely to be ablated, the clinical presentation of these tachycardias, alternatives to ablation, the relative potential for success or complications, and modifications of the procedure that might reduce the risk of ablation in this group.

## Tachycardia Substrates in Small Children

**Figure 1:**
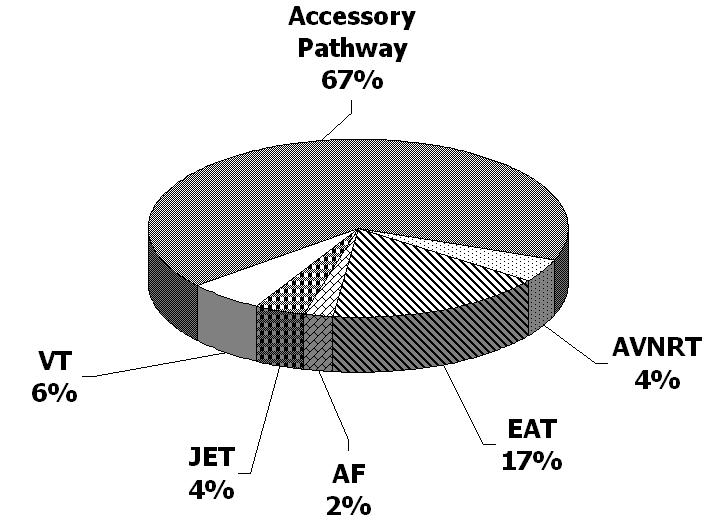
Distribution of substrates for infants (< 1.5 yrs) undergoing RFA The majority of infants who have undergone RFA have had an accessory pathway-mediated tachycardia, such as AVRT. The next most common substrate ablated was EAT. AVNRT, AF, JET, and VT were ablated in a similar number of patients. This distribution is similar to the distribution of substrates diagnosed in children of this age. AVNRT = atriventricular reciprocating tachycardia, EAT – ectopic atrial tachycardia, AF = atrial flutter, JET = junctional ectopic tachycardia, VT = ventricular tachycardia.  *Data from reference [2]*

It is necessary to have a clear understanding of which tachycardias that are likely to be ablated in small children prior to adequately discussing whether or how to ablate them.  Atrioventricular reciprocating tachycardia (AVRT) is the most common type of SVT in small children [9] with a prevalence of approximately 0.1-0.15% [10]. AVRT, AV nodal reentrant tachycardia (AVNRT), and ectopic atrial tachycardia (EAT) respectively account for 80%, 5%, and 15% of SVT in children less than 1 year of age and approximately 65%, 25%, and 10% of SVT in children who are between 1 and 5 years [9]. Atrial flutter (AF) is relatively uncommon in this age group.  Congenital junctional ectopic tachycardia (JET) is rare.  However, JET can also be uncommonly encountered in the small group of neonates who undergo neonatal surgery for CHD.  Although the true prevalence of ventricular tachycardia (VT) in small children is unknown, it is felt to be relatively uncommon.  Thus far, the distribution of substrates ablated in infants less than 1.5 years of age has been shown to be similar to the relative prevalence of the tachycardias mentioned above (Figure 1) [2].   Since AVRT is the most likely tachycardia to be encountered and ablated in small children, the remainder of this discussion will focus primarily, but not exclusively, on AVRT with or without associated preexcitation.

## Clinical Presentation of Tachycardias in Small Children

There are few true “natural history” studies for tachycardias that present in childhood as there has been a great propensity to treat once the problem has been identified.  However, several important observations can be made with regard to the course of these tachycardias.

Approximately, 1/3-2/3 of patients who present with WPW or AVRT in infancy will not have a recurrence of tachycardia after medication is discontinues at their first birthday [5,6].  When tachycardia does recur, it is usually well tolerated.  However, there are occasions when medical intervention is not sought for a prolonged period of time resulting in hemodynamic collapse [11].  Mortality rates of approximately 5% have been reported in infants with WPW and AVRT [6,7].  While some of these deaths could be attributed to medication issues, it is likely that others were do to hemodynamic collapse after the development of tachycardia-induced cardiomyopathy and to cardiac arrest secondary to rapid ventricular conduction over the accessory pathway during atrial fibrillation in patients with WPW. Tachycardia-induced cardiomyopathy is a known consequence of prolonged tachycardia in infants and needs to be distinguished from myocarditis and recognized as a curable cause of cardiomyopathy [12] The risk of sudden death in WPW is approximately 0.1% per year overall and may be as high as 0.6% per year in “high risk” patients [13]. Klein et al originally reported the association between antegrade accessory pathway conduction properties and ventricular fibrillation in patients with WPW [14].  Although the capability for a young child’s heart to sustain atrial fibrillation has been debated, sudden death has been reported in the pediatric population and has been the presenting symptom in 2.3% [15].  The risk of sudden death in children with WPW has been associated a preexcited R-R interval of 190-220 msec during atrial fibrillation induced at EPS (sensitivity of 100% and a specificity of 72-74%) [16]. It is important to note that as the child ages, the conduction properties of the AV node and the accessory pathway will also change, so that assessing risk is somewhat of a moving target.  Despite this, the vast majority of patients with WPW and AVRT will not experience severe symptoms.

There has been less published on the natural history of other tachycardia substrates.  VT has a higher likelihood of causing acute hemodynamic collapse.  While EAT is less likely to cause acute hemodynamic compromise, its incessant nature increases the risk of developing tachycardia-induced cardiomyopathy.  Any of these tachycardias are less likely to be tolerated in children with structural heart disease.

## Alternatives to Catheter Ablation

There are three alternatives to catheter ablation.  These are not treating, treating with drugs, and performing surgical ablation.  Surgical ablation is a precursor to catheter ablation and is much more invasive so is rarely still performed today except in the form of an atrial maze procedure being more commonly performed for more complex arrhythmias in older patients and often with concurrent hemodynamic structural surgical intervention, thus it will not be discussed further.  The other options are still practiced but are based on limited data.

### No Treatment

The decision not to treat patients with WPW carries the potential risks of SVT recurrence or sudden death.  The use of transesophageal pacing studies have been shown to have a negative predictive value of 74-100% for predicting SVT recurrences [17,18].  This method can also be used to establish the likelihood of recurrent AVNRT or to determine the conduction properties of an accessory pathway while assessing the risk of sudden death in children with WPW.  Thus, if the patient is not at risk for sudden death and is not inducible, no treatment becomes an option.  However, other factors must be considered, such as access to medical care and parent comfort and abilities to handle recurrences.

No treatment strategy for other arrhythmias is less established.

### Medical Treatment

Although there have been a great number of publications on the medical management of tachycardias in small children, there have been no controlled studies.  Most studies report limited success of drugs to control SVT.  Success rates for digoxin or beta-blockers have been reported to be approximately 50% while success for the more toxic class I and class III agents are not much better [18]. Although various combinations may increase success, they also increase the potential for side effects, particularly when class I and class III agents are combined. Reports of more aggressive drug combinations have been limited to a very small number of patients and thus their safety is essentially unknown.  It is clear, however, that drug therapy, whether given as single agent or in combination, has the potential for adverse reactions including death [15].   While most drug therapies will be well tolerated, the lack of controlled data delineating their efficacy, makes balancing the risk/benefit ratio for drug therapy difficult.  Another point about drug therapy is that it is unlikely to protect one against rapid conduction during atrial fibrillation in patients with WPW unless they are being treated with class I or class III drugs.

## RF Ablation

### Success

Several studies on RFA in children have shown that there is no difference in success rate in small children for eliminating arrhythmias on the whole or AVRT in comparison to older children [2-4].  The comparable success rates may be partially due to the fact that ablations in smaller children are more likely to be attempted by more experienced pediatric electrophysiologists [2] and experience has been shown to be an important factor in  successful pediatric RFA procedures [19]. Although pediatric ablation registry studies involving the entire pediatric age span have found lower success rates in children with structural heart disease [3,4],  substrate elimination in infants has been shown not to be influenced by the presence of structural heart disease [2].  Thus, beliefs that RFA will be less successful for infants with heart disease are incorrect and should not deter attempts in those infants in whom RFA is indicated.  Another interesting difference between infants and older children is that infant accessory pathway elimination may not necessarily be related to AP location [2,4].  Similar to reports in adult, the presence of multiple accessory pathways in infants is associated with lower success rates.

### Complications

#### Overall

As stated previously, children weighing less than 15 kg have been shown to be at increased risk for complications during RFA [3] [4].   In Blaufox et al.’s pediatric ablation registry study of infants less than 1.5 years of age, a higher complication rate was found in infants in comparison to older children, but power limitations may have prevented the difference from reaching statistical significance [2].   When data from 231 registry patients weighing < 15 kg but being > 1.5 years old were factored in to the analysis, this study did confirm a higher complication rate in children < 15 kg.  However, there was no appreciable difference in major complications for the infants less than 1.5 years of age.

Typically, RF lesions made in vivo, vary in size from non-existent to 5 or 6 mm radius. The average adult heart has a wall thickness of 3-12 mm’s.  However, the size of the heart and its internal structures are proportional to body size [20]. Consequently, the theoretical risk of injuring cardiac structures in small children is higher and might depend specifically on the parameters that influence lesion size. In controlled animal studies, RF lesion size is directly related to catheter tip size, RF power, tip temperature and lesion duration [21]. Further, more RF applications is clearly more likely to increase total lesion volume.  Similarly, repeated thermal injury in nearby areas can be expected to increase the chance of injury to adjacent vital structures, again with an inverse relation to patient size.  Finally, the scars created by RF energy have a greater chance of expanding into vital structures when the myocardium is less mature [22].  As the greatest increase in heart size and maturity occurs during the neonatal and infant ages, disparities in size and myocardial maturity appear to be important even within the subgroup of small children.

The major complications in small children include pericardial effusion, pneumothorax, AV block, and death [2].  In addition to these complications, small children may be at particular risk for coronary artery injury.

#### Death

The overall mortality associated with pediatric RFA has been reported by Schaffer et al. as 0.12% [23]. This study contained the report of an infant with a structurally normal heart who died 2 weeks following RFA for AVRT.  Approximately 111 RFA procedures were done infants during the period covered by Schaffer et al yielding an infant mortality of approximately 0.9%.  In addition, Schaffer’s study reports the death of an 18 month old child with congenital heart disease who underwent RFA and died the following day with fever and hypotension, but in whom no link between death and RFA could clearly be established.   Blaufox et al reported the acute death of a separate infant yielding an infant mortality of 0.74% in that study.  The discrepancy between Schaffer et al. is due to the difference in time periods covered by 2 studies and the fact that the Blaufox et al. report represented only acute results while the Schaffer et al. report included follow up.   Although each report only includes one instance of death and thus the actual incidence might be somewhat inaccurate, it is evident that death can result from infant RFA.

#### AV Block

**Figure 2:**
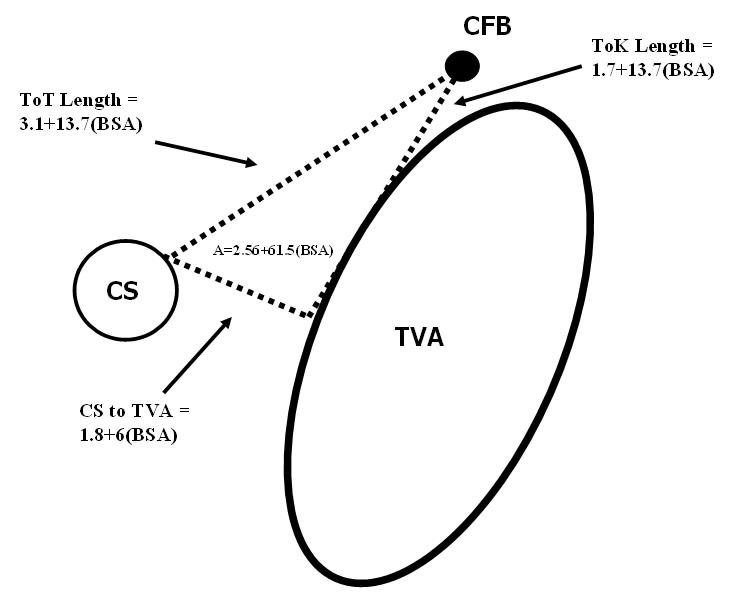
Dimensions of the Triangle of Koch This drawing illustrates the relative dimensions of Koch’s Triangle in children. Each side and the area of the triangle are proportional to body size according to the formulas shown. CFB = central fibrous body, CS = coronary sinus, TVA = tricuspid valve annulus, ToK = Triangle of Koch, ToT = Tendon of Todaro, A = Triangle of Koch area. *Data from reference [24]*

Body weight less than 15 kg is an independent risk factor for AV block during RFA [4]. Similar to reports in older children [4], RFA for septal AP’s in infants is also associated with a higher incidence of heart block [2]. Thus, the ablation of septal substrates in small children is particularly risky.  This is not surprising if one considers the relative sizes of RF lesions and the triangle of Koch in children.  As stated previously, RF lesions generally have a radius of 5-6mm.  Unlike in adults, the dimensions of the Triangle of Koch are proportional to body size in children (Figure 2) [24].  Therefore, a lesion with a fixed size will have a greater likelihood of injuring vital structures within and around the triangle of a smaller child.   So, great caution must be used when approaching these substrates.

#### Coronary Injury

 Coronary artery injury during RFA is a rare, but serious event [25] that has occasionally produced death [23,26].  Nearly all of the reports of coronary artery injury following RFA have been single case reports that have involved accessory pathway elimination.  Of the 5 deaths in children with structurally normal hearts reported by the pediatric ablation registry [23], 1 was the result of thermal injury to the left main coronary artery and subsequent thrombosis of that vessel in a 13 year old child who underwent RFA for AVRT.  In addition to these reports involving accessory pathways, there has been one report of injury to the posterior left ventricular branch artery during slow pathway ablation for AVNRT in a 15.5 kg child [27].

The mechanism of coronary injury is likely to be a combination of direct thermal injury and subsequent inflammatory response.  The inflammatory component of tissue injury caused by RF energy has been shown to invade layers of the right coronary artery, leading to acute narrowing when RF energy is applied to the atrial side of the lateral tricuspid annulus in pigs [28].  Further maturation of this injury can result in significant late coronary stenosis [29].  Thus, with RF energy application, coronary stenosis may occur acutely or may be delayed.

In addition to the potential for coronary injury to be delayed, it may also be subtle, thus it may go unrecognized so that the incidence of sub-clinical coronary injury is likely to be underestimated. Blaufox et al. presented a patient in whom coronary injury was nearly missed because ST segment changes did not occur until 100 seconds after the last RF application and resolved spontaneously within minutes despite a significant persistent stenosis of the posterior left ventricular branch coronary artery [27].  In large retrospective and prospective studies where there were no coordinated attempts to investigate coronary injury after RFA, the reported incidences of injury were 0.03% in children [4], and 0.06-0.1% in adults [30].  However, in a study where coronary angiography was performed before and after RFA for accessory pathway-mediated tachycardias, Solomon et al reported a 1.3% incidence of coronary artery injury in 70 patients following RFA for accessory pathway-mediated tachycardias [31]. Thus, unless evidence for coronary artery injury is actively sought, it may go undiagnosed and underreported.  With the exception of severe stenoses, injury may go unrecognized until premature coronary disease becomes associated with people who have undergone RFA as young children.

### Modifications

**Figure 3:**
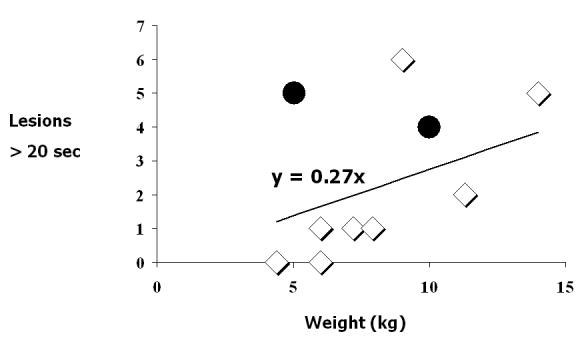
Relationship between RF lesions, weight and complications This graph shows a higher likelihood of complications when the ratio between RF lesions (duration > 20 seconds) and body weight increases.  The formula y = 0.27x is the trend line for this ratio in patients without complications. Although the data is based upon a few patients and has not been formally tested, it illustrates the main general message to limit RF lesion number and duration based upon patient weight. *Data from reference [11]*

Modifications to the standard RFA procedure, such as, the use of smaller caliber catheters with smaller tips, the use of 5-second applications with lower temperature set points to test location accuracy, and the limitation of full applications to 20 seconds have been proposed and implemented [32,33].  Although these modifications are based upon physical and animal studies of the effects of radiofrequency energy on the maturing myocardium, aside from limiting the number of RF applications, which has been shown to decrease mortality [23], little clinical data exists to support these modifications in the application of RFA in small children [2,11,32,33].  However, for AVRT, Blaufox et al reported that the relationship between complications and application number and duration holds up for applications with durations greater than 20 seconds only when the number of these applications is indexed to body weight in kg (Figure 3) [34]. In other words, the increase in risk of giving applications with a duration > 20 seconds was proportional to the patient’s weight.  Inherent in the idea of limiting the number of lesions, is the abstention from giving an “insurance” lesion.  In addition to limiting the length and number of applications based upon the patients size, perhaps the most important modification proposed in this study is the lowering of one’s threshold for accepting failure, for numerous studies have shown that a greater number of lesions will be given during a failed procedure in comparison to a successful one [2].

## Alternative Energy

An alternate strategy for catheter ablation is to find an energy source that may be safer than RFA in small children.  Although there have been no trials of cryoablation in small children, there are several aspects to this technology that make it a potential alternative.  The results of prior animal studies suggest that some advantages of cryo-therapy may be particularly important for children.  Cryo-ablation sites are histologically well delineated, discrete, and show homogeneous dense fibrous tissue without viable myocardium interspersed [35].  Cryo-lesions are smaller than RFA lesions [36], contributing to the ability to safely create cryo-lesions even adjacent to the His bundle.  In addition, both cryoablation allows for reversible loss of tissue function [37] [38].  These transient effects occur for both the normal AV conduction fibers and the targeted tachycardia substrate.  Friedman et al [38]. also reported 12 instances of transient AV block, 11 of which occurred during cryo-ablation modes, and all of them resolved completely.  Because the leading edge of the ice ball during cryo-therapy is by definition near 0 °C and warmer than the temperature measured at the catheter tip, it is likely that discontinuation of cryo-therapy at the first signs of an electrophysiological effect will reverse that effect.  Another potential safety feature of cryo-therapy is catheter stability at the point of tissue freeze.  This lack of tip movement should both improve success when the catheter is in the correct place, and prevent lesion spread to undesirable locations through the sliding movement seen with RFA.

Because cryo-lesions are more delineated and smaller than RF lesions, cryoablation may require more precise positioning, particularly for the relatively discrete accessory pathways.  Therefore it is not surprising that the acute success rate may be lower.  Friedman et al reported an overall success rate of 69% for AVRT in adults [38].  However, success rates for septal accessory pathways and AVNRT were more comparable to those with RF ablation [38].   There is limited data with cryoablation in children, but our own experience with cryoablation in pediatric patients supports the data found in adults.  We have experienced success rates of 96% for AVNRT and 63% for AVRT without any major complications. All instances of AV block have been transient with full recovery within a few seconds. Although our experience is limited, given the potential safety advantages for this technology, it is reasonable to consider using it prior to RF ablation in small children despite the lower expectations for success.

## Indications

**Figure 4:**
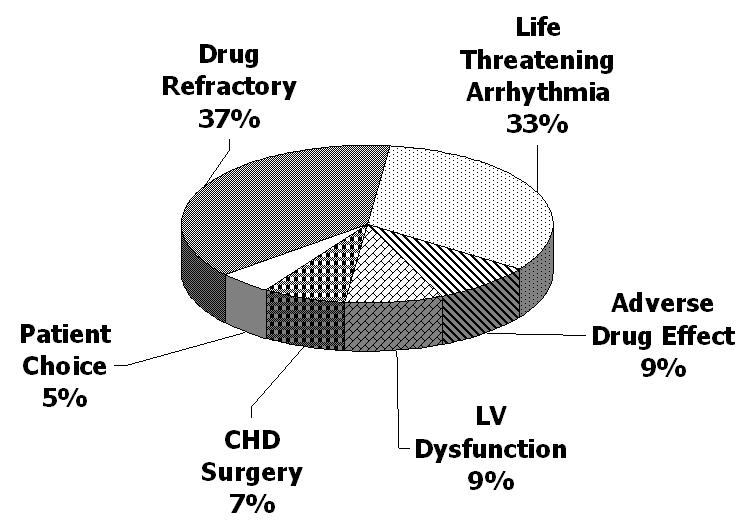
Distribution of indications for infants undergoing RFA as reported from the Pediatric Radiofrequency Ablation Registry Infants who have undergone RFA have done so because they have been perceived to be at greater risk from their arrhythmia or more difficult to treat.  This is in contrast to older children in whom RFA has been done for “patient choice” 51 % on the time (data not shown). *Data from reference [2]*

Infants who have undergone RFA have done so for indications that are different than those for older children in whom RFA is done for “patient choice” 51% of the time. (Figure 4) [2].   The differences in indications between infants and older children demonstrate that infants are sicker upon presentation and perceived to be at greater risk during arrhythmia.   Although these perceptions are heightened for infants with structural heart disease and there is a higher incidence of structural heart disease for infants undergoing RFA, the incidence of structural heart disease does not entirely account for these perceptions because they are still true for infants with structurally normal hearts [2].

Because the definition of indications reported from the pediatric RFA registry, such as refractory to medical therapy, vary widely from center to center, others have sought to establish more clear cut indications.  In 2002, a position statement was published by members of the Pediatric Electrophysiology Society and endorsed by the North American Society of Pacing and Electrophysiology. (Friedman RA NASPE)  *Class I* indications, in which there is clear and consistent agreement that RFA will benefit the patient, included:1) WPW following aborted sudden death, 2) WPW and syncope with a shortest prexcited R-R < 250 msec, 3) chronic or recurrent SVT with ventricular dysfunction, 4) and recurrent VT associated with hemodynamic compromise and is amenable to RFA. *Class IIA* indications, in which the majority of opinion or data favor RFA, include 1) recurrent and/or symptomatic SVT refractory to medical therapy and age > 4 years, 2) impending congenital heart surgery when vascular or chamber access may be limited following surgery, 3) chronic (>6 months) or incessant tachycardia with normal ventricular function, 4) chronic or frequent recurrences of intraatrial reentrant tachycardia, and 5) palpitations with inducible SVT during EPS.  *Class IIB* indications, in which there is a clear divergence of opinion regarding the need RFA, include: 1) asymptomatic WPW and age > 5yrs when the risk/benefits of RFA have been explained to the family, 2) SVT, age > 5 yrs, as an alternative to chronic medical therapy that has controlled the tachycardia, 3) SVT, age < 5 yrs, when medications, including sotalol and amiodarone, have not controlled the tachycardia or have resulted in intolerable side effects, 4) intraatrial reentrant tachycardia, 1-3 episodes per year requiring medical intervention, 5) AV node ablation for intratrial reentrant tachycardia, 6) one episode of VT with hemodynamic compromise and amenable to RFA.  *Class III* indications, in which there is agreement that RFA is not indicated, include: 1) asymptomatic WPW, age < 5 yrs, 2) SVT, controlled with medication, age < 5 yrs, 3) Nonsustained and non incessant VT without ventricular dysfunction, 4) Nonsustained, asymptomatic SVT.

## Conclusion

Catheter ablation in small children should be reserved for truly life threatening or refractory arrhythmias after multiple failed attempts at medical management, which may include various combination therapies.  RFA should be performed by an experienced pediatric electrophysiologist who undertakes various strategies to reduce risk, including limiting power and temperature as well as application duration and attempts based upon the patient’s size. Consideration of the use of alternate sources of energy like cryoablation prior to RFA may be helpful.  Despite a high potential for success, having a lower threshold for accepting failure is essential.
